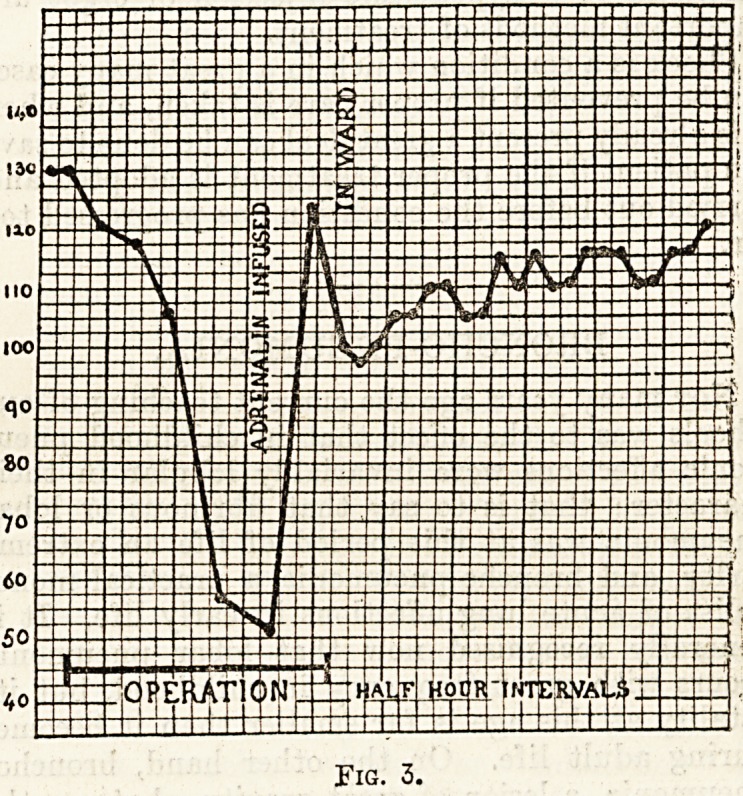# The Treatment of Shock and Collapse Following Surgical Operations

**Published:** 1906-05-26

**Authors:** P. Lockhart Mummery

**Affiliations:** Hon. Surgeon, King Edward VII. Hospital for Officers; Assistant Surgeon, St. Mark's Hospital for Diseases of the Rectum; and Surgeon to Out-Patients, North Eastern Hospital for Children


### HOSPITAL CLINICS.

### THE TREATMENT OF SHOCK AND COLLAPSE FOLLOWING SURGICAL OPERATIONS.

By P. LOCKHART MUMMERY, B.C., F.R.C.S., Hon. Surgeon, King Edward VII. Hospital for Officers; Assistant Surgeon, St. Mark's Hospital for Diseases of the Rectum; and Surgeon to Out-Patients, North Eastern Hospital for Children.

ONE of the chief dangers of severe and prolonged operations is the occurrence of surgical shock, either coming on during the operation or some short time after the patient has been put back to bed. Every-one who has any experience of surgical cases knows the condition of shock, so I will not waste time by a description of the symptoms. It will be enough to say that the patient's skin is blanched, cold to the touch, and often covered with beads of perspiration which makes it feel what is often called "clammy." The pulse is weak and feeble, and in bad cases almost imperceptible at the wrist, the respiration is shallow and hurried, and there are dark lines under the eyes, while the lips are often of a bluish colour.

This condition of shock may result from almost any operation, but is more liable to occur after certain kinds of operations and in certain types of patient.

It will not be out of place, I think, to briefly consider the causes of this condition of shock, as it is only by understanding the causes at work that we can hope to treat the condition properly and effectually. Shock results from a breakdown in the vascular hydraulic mechanism which controls and maintains the blood-pressure at a more or less constant level during health. The blood-pressure is normally maintained by the contraction or relaxation of the blood-vessels, and this mechanism is controlled by the vaso-motor centres in the medulla at the base of the brain, and by some other vaso-motor centres in the upper part of the spinal cord.

In surgical shock these centres have become exhausted from the strain which has been put upon them in attempting to keep up the blood-pressure during and immediately after the operation. And of course it must be understood that a severe injury, such as will result from an accident, acts in the same way as an operation; for after all an operation is only an intentional injury.

The result of the vaso-motor centres having become exhausted or tired is that the blood-pressure in the blood-vessels falls much below the normal level, and this fall in the blood-pressure after operations can be seen or measured by a suitable instrument called a sphygomometer. Fig. 1 is a chart of the blood-pressure in a case of shock followed by recovery, and it can easily be seen how great a fall in pressure has resulted from the operation.

It is very important to bear in mind the fact that the cause of shock is exhaustion of the nerve centres. Our common sense tells us that when a person or animal is suffering from exhaustion the proper thing —in fact, the only thing—which can do any good is to give them rest until they have recovered. No one with any common sense would think of flogging a horse which had fallen down from exhaustion; yet curiously enough this is too often the principle on which shock is treated. The vaso-motor nerve centres of a patient who is suffering from shock are in the condition of the exhausted horse which has fallen down, and it is just as reasonable to flog the horse as to try and stimulate these centres.

The old-fashioned way of treating shock is to inject strychnine or some other stimulant, such as ether, into the patient's arm; now the effect of this treatment is simply to stimulate the exhausted centres, and comes to just the same thing as flogging the horse. You may succeed in stimulating the

FIG. 1.

centres just as if you hit hard enough you may make the horse get up, but the exhaustion will only be made worse later on.

This is not just an idea, or what is often called a theory, but is a fact which has been most carefully proved by experiments and by careful observations; and it cannot be too strongly insisted upon that it is absolutely worse than useless to administer strychnine or any stimulant to a patient suffering from shock. It not only does no good, but it tends to increase the severity of the condition.

A very great deal may be done to prevent shock resulting from an operation, and this is by far the best method of combating this very grave condition.

The preparation of a patient for operation must be carried out judiciously, as if it be overdone the tendency for shock to result after the operation may be much increased.

The usual practice of purging and starving a patient previous to an operation must be carried out with caution. Free purgation greatly lowers the patient's resisting power to shock, and starvation has the same effect.

This is particularly the case with old people and children, and it is advisable not to starve such patients previous to any serious operation. A little easily digested liquid food, such as milk or milk and egg, may be given an hour or two before an operation with safety and without any risk of causing vomiting during the operation. In young children it is safer not to interfere with the feeding at all, except to see that only easily digested and liquid food is given for six hours before operation. To make a child go breakfastless before an operation in the middle of the day is wrong, and will greatly increase the risk of shock; and the same may be said of old people. Before any serious operation on an elderly person or a child the bowels should be well opened two or three days prior to the operation, and then kept open each day by means of an ordinary enema, while at the same time a diet should be given which, while possessing a maximum amount of nutritious value, will leave but little solid residue.

Children and old people should also have the limbs kept warm during an operation by wrapping them in cottonwool before the operation, or by the wearing of thick woollen socks and armlets. Care should also be taken to see that the operating-table itself is warmed, either by filling the tanks (in the case of a table so provided) with warm water or by placing a large hot-water bottle beneath the blankets. It is advisable to remember, however, that patients in a state of shock can be burnt or blistered by a heat which would be quite insufficient to cause damage under ordinary conditions.

After an operation much may be done to prevent or relieve shock by simple means. The patient should be got back to bed as quickly as possible and without undue jolting.

The pillow should be removed so that the patient lies quite flat in the bed, and the foot of the bed should then be raised so that the abdomen of the patient is at a slightly higher level than the head and shoulders. It is, of course, important that the patient should be kept warm by blankets and hot-water bottles, but the greatest care must be taken that there is nothing to interfere with the movement of the chest during respiration; the clothing should be quite loose, and all bed-clothes should be well supported on a cradle so as to take all the weight off the body. This is very important in young children.

A hypodermic injection of morphia of a $\frac{1}{4}$ to a $\frac{1}{3}$ of a grain, quite independently of whether pain is complained of or not, is a most valuable means of treating shock. Much may often be done to prevent shock by a morphia injection just previous to the operation, and morphia, by quieting the nervous system and preventing the transmission to the brain of painful sensory stimuli, will do a great deal to relieve shock after an operation. I believe morphia to be one of the most valuable drugs which we possess for this purpose. The importance of nourishment must not be forgotten—as it too often is—in the treatment of patients suffering from shock. Nourishment is of more importance than drugs, and should be given in some form or another as soon as possible. It is difficult to administer

Fig. 2.—A Fatal Case of Shock, showing the Fall in Blood Pressure, resulting from a Serious Operation—Excision of the Uterus for Fibroids.

nourishment just after an operation in the usual manner, as it is apt to cause vomiting; but nutrient enemata can almost always be given at once, and milk or egg albumen can usually be given by the mouth very soon after operation. Subcutaneous feeding with sterilised olive oil is also a valuable means of administering nourishment in suitable cases. Sugar, especially in the form of grape sugar, which can easily be made by stewing raisins, is also a valuable form of nourishment, and has the advantage that it needs no digestion, and so is at once available to supply the tissues. It can be given either by the mouth or rectum.

Very young children should be given the bottle directly after the operation. Infants will often take milk directly they are conscious after the anæsthetic, and are seldom sick. I always let a young child have the bottle directly it is back in the ward, and in most cases the nourishment is taken readily, and no sickness results. Infants especially will not stand starving for even a few hours, and do best when the feeding is interfered with as little as possible. In one case an infant six months of age, whom I had operated upon for intussusception was given the breast within 20 minutes after the operation was over. The child went to sleep afterwards, and recovered without any unpleasant symptoms.

I have gone rather thoroughly into this question of feeding after an operation as I think it is a very important factor in combating shock, and one which is often overlooked.

A useful method of treating shock in suitable cases is by bandaging the abdomen; this acts by reducing the capacity of the peritoneal cavity and so preventing the blood from accumulating in the vessels of the intestines and viscera—the so-called splanchnic area. The bandage, to be effective, must be tight, and a flannel binder or soft towel wrapped tightly round the abdomen and secured by safety-pins is the best form of bandage for the purpose. It must not be applied in such a way as to embarrass the chest movements or it will do harm by restricting respiration.

Saline infusion into the veins is a most useful method of treating shock, but to be really effective it must be used early and not resorted to only when the patient is "in extremis" as is too often done. This is sometimes incorrectly called transfusion, but intravenous infusion is a better term. Transfusion means taking the blood from one patient and putting it into the veins of another, a practice which is now quite obsolete. The fluid used for intravenous infusion should, if possible, be made up by dissolving tablets of physiological-salt solution (of which there are now several good preparations) in warm sterilised water at a temperature a little above the normal blood temperature.

The canula of the apparatus must be inserted into a big vein in the arm and the fluid slowly allowed to flow in from a funnel attached to the canula by a rubber tube, care being taken to see that no air gets in. About two pints of this fluid may be allowed to run in at a time and the process repeated if the improvement is not maintained. Another method, which is very popular on the Continent, is to infuse the fluid under the skin, usually of the axilla or breast. It can also be given by the rectum. But in bad cases of shock the intravenous infusion is to be preferred.

The extract of the supra-renal gland and its derivatives, such as adrenalin, has a very powerful action in raising the blood-pressure, and this drug acts directly upon the vessel-walls and not through the vaso-motor centres, so that in it we have a means of directly raising the blood-pressure in cases of shock, independently of the exhausted nerve centres. To be effective this drug must be infused directly into the veins. It should be used only in dilute solutions of 1—20,000 with salt solution, as its action is very rapid and powerful. It is of the utmost value in grave cases of shock and may save life when nothing else can. Unfortunately its action is transitory, and to be effectual in bad cases, the infusion must be repeated at short intervals until the shock has passed off. Fig. 3 shows the chart of the blood-pressure in a case of shock treated by the infusion of adrenalin.

Hypodermic injections of ergot, a drug which acts in a somewhat similar manner to the extract of supra-renal gland, seems to be of value in the treatment of conditions of lowered blood-pressure, such as shock. The chief difficulty hitherto has been that the preparations of this drug could not be relied upon. There are now, however, one or two preparations of ergot on the market which seem to give fairly uniform results, and the drug is worthy of a more extended trial in the treatment of shock.

There is one kind of shock which occurs after surgical operations which must be mentioned, it is often called "deferred shock." It is most often

Fig. 3.

seen in children, but is not uncommon at all ages. What happens is that the patient gets through the operation well and everything seems quite satisfactory for the first few hours after the operation. Then, about four or five hours after the operation, and usually after consciousness has returned, symptoms of shock are noticed and the patient quickly sinks into a dangerous condition of shock. This is a very grave condition and calls for prompt treatment. This "deferred shock" should, however be prevented, and much may be done to combat it by careful attention to the patient after the operation. In children it is best always to treat the patient afer any serious operation as if shock were present, even though there may be no symptoms of that condition.

Thus the foot of the bed or cot should be always well raised and the patient kept warm. What has already been said with regard to the administration of nourishment is of the greatest importance in this connection, and nourishment properly administered will do much to prevent the onset of this form of shock.

The administration of morphia after the operation is also a most valuable method of preventing the onset of deferred shock.

I will conclude this article by giving a brief summary of the points which it is important to observe in the treatment of shock:—

1. The use of stimulants in any form must be avoided.

2. Nourishment in some form or another must be administered as soon as possible.

3. A hypodermic injection of a small dose of morphia should be given.

4. The foot of the bed should be well raised and everything possible done to increase the bloodpressure in the vital parts of the circulation, namely the chest and head.

5. The intravenous infusion of saline solution should be performed as soon as symptoms of shock are noticed.

6. Intravenous infusion with a weak solution of adrenalin or a hypodermic injection of ergot are important methods of treatment.

Shock is a condition which in a great many cases can be prevented if proper care is taken, and when it is already present a great deal can be done to save the patient if the proper treatment is adopted and carried out before the condition has progressed too far.

## Figures and Tables

**Fig. 1. f1:**
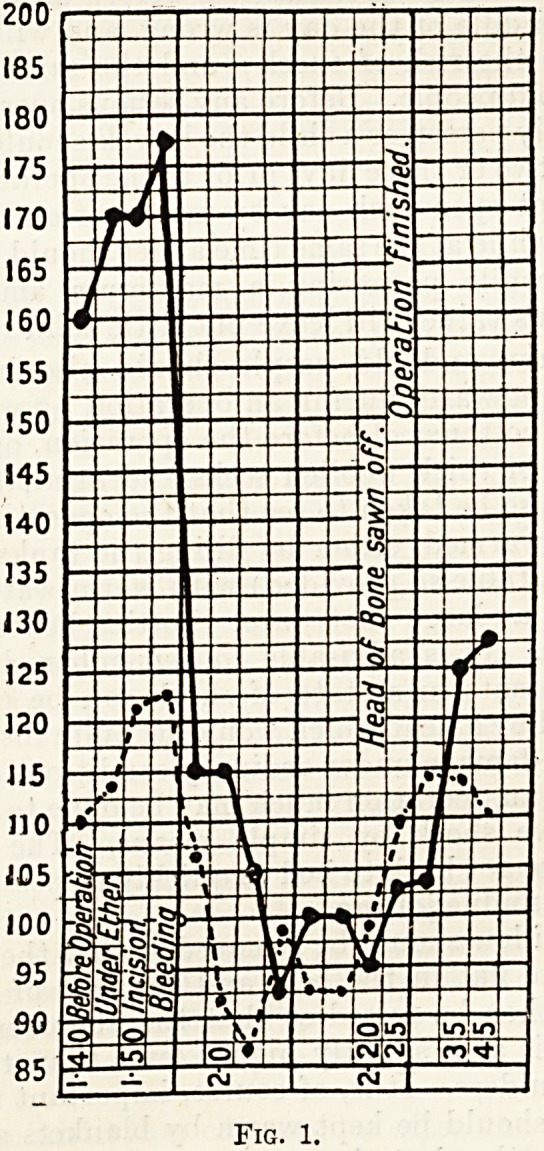


**Fig. 2. f2:**
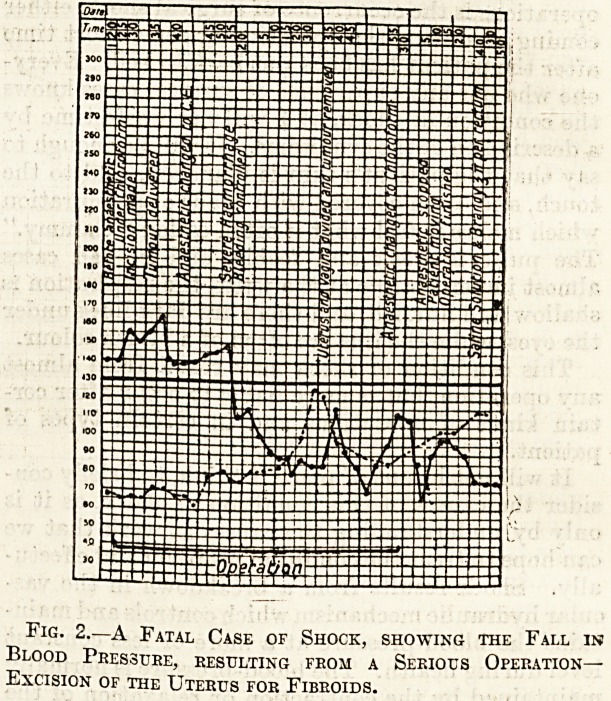


**Fig. 3. f3:**